# Coordination between p21 and DDB2 in the Cellular Response to UV Radiation

**DOI:** 10.1371/journal.pone.0080111

**Published:** 2013-11-18

**Authors:** Hao Li, Xiao-Peng Zhang, Feng Liu

**Affiliations:** National Laboratory of Solid State Microstructures and Department of Physics, Nanjing University, Nanjing, China; Uni. of South Florida, United States of America

## Abstract

The tumor suppressor p53 guides the cellular response to DNA damage mainly by regulating expression of target genes. The cyclin-dependent kinase inhibitor p21, which is induced by p53, can both arrest the cell cycle and inhibit apoptosis. Interestingly, p53-inducible DDB2 (damaged-DNA binding protein 2) promotes apoptosis by mediating p21 degradation after ultraviolet (UV)-induced DNA damage. Here, we developed an integrated model of the p53 network to explore how the UV-irradiated cell makes a decision between survival and death and how the activities of p21 and DDB2 are modulated. By numerical simulations, we found that p53 is activated progressively and the promoter selectivity of p53 depends on its concentration. For minor DNA damage, p53 settles at an intermediate level. p21 is induced by p53 to arrest the cell cycle via inhibiting E2F1 activity, allowing for DNA repair. The proapoptotic genes are expressed at low levels. For severe DNA damage, p53 undergoes a two-phase behavior and accumulates to high levels in the second phase. Consequently, those proapoptotic proteins accumulate remarkably. Bax activates the release of cytochrome c, while DDB2 promotes the degradation of p21, which leads to activation of E2F1 and induction of Apaf-1. Finally, the caspase cascade is activated to trigger apoptosis. We revealed that the downregulation of p21 is necessary for apoptosis induction and PTEN promotes apoptosis by amplifying p53 activation. This work demonstrates that how the dynamics of the p53 network can be finely regulated through feed-forward and feedback loops within the network and emphasizes the importance of p21 regulation in the DNA damage response.

## Introduction

The tumor suppressor p53 has a critical role in maintaining genome integrity by mediating the cellular response to various stresses including DNA damage [1]. p53 mainly functions as a transcription factor, regulating expression of target genes to elicit multiple cellular processes including cell cycle arrest and apoptosis [2]. The promoter selectivity of p53 depends heavily on its concentration, posttranslational modification, and interaction with cofactors [3]. Moreover, p53 can translocate to mitochondria, activating the proapoptotic proteins like Bax to induce apoptosis in a transcription-independent manner [4].

It was experimentally shown that the concentration of p53 undergoes pulses upon ionization radiation in MCF7 cells [5]. Since then many theoretical models have been developed to clarify the dynamic mechanism for p53-mediated cell-fate decision following DNA damage. Zhang *et al.* and we proposed that the cell fate can be determined by the number of p53 pulses, which depends on the severity of DNA damage [6], [7]. We further proposed that p53 may exhibit two-phase dynamics in MCF10A cells expressing PTEN (phosphatase and tensin homolog) normally [8]. Primarily phosphorylated p53, whose concentration undergoes pulses with low amplitudes, induces cell cycle arrest in the early phase of the response, while fully phosphorylated p53 accumulates to high levels and triggers apoptosis in the late phase of the response to severe damage. We recently revealed how two antagonistic cofactors of p53, Hzf and ASPP1/2, interact to affect cellular outcome [9]. Of note, few models have been built to explore the cellular response to ultraviolet (UV) radiation. It is intriguing to probe how the UV-irradiated cells make a decision between survival and death.

p21 is a transcriptional target of p53 [10]. It mainly acts as an inhibitor of cell cycle progression by inhibiting the activity of cyclin-dependent kinase (CDK)-cyclin complexes and proliferating cell nuclear antigen (PCNA) [11]. On the other hand, the prosurvival function of p21 has recently attracted wide attention [12]. Several mechanisms for apoptosis inhibition by p21 have been reported. p21 can protect cells from apoptosis by directly binding and inhibiting procaspase-3 [13]. It can block apoptosis downstream of mitochondria by repression of CDK-mediated caspase-9 activation [14]. p21 can also prevent apoptosis by suppressing the transactivation of some transcription factors like c-Myc [15], Thus, downregulation of p21 is required for apoptosis induction. However, the dynamic mechanism underlying p21 downregulation is still less understood.

When cells are exposed to UV radiation, the major forms of DNA damage are cyclobutane pyrimidine dimers (CPDs) and 6–4 pyrimidine-pyrimidone photoproducts (6–4 PPs), which cross-link adjacent DNA bases [16]. Such DNA damage can be fixed through nucleotide excision repair (NER) [17]. Due to DNA excision in the NER, single-strand breaks (SSBs) are formed during the first 15–60 min after irradiation and are repaired slowly during the next few hours [18]. SSBs can be detected by the ATR (ataxia telangiectasia mutated and Rad3-related) kinase [19]. The damaged-DNA binding protein 2 (DDB2) has a very high affinity for UV-damaged DNA and participates in the NER by recruiting the NER factor XPC [20]. In human cells, DDB2 is directly induced by p53 upon DNA damage [21]. DDB2 also acts as a substrate-adaptor for the E3 ubiquitin ligase Cul4-DDB1 [22]. DDB2 promotes the degradation of p21 after UV radiation, thereby releasing cells from the apoptosis inhibition by p21 [23]. This raises the issue of how p21 activity is tightly modulated to allow for cell cycle arrest or apoptosis.

Motivated by the above considerations, we developed a four-module model of the p53 signaling network responding to UV-induced DNA damage. We considered five target genes of p53: Mdm2, p21, DDB2, PTEN and Bax. The Rb/E2F1 pathway and the caspase cascade downstream of cytochrome c release were also included. We found that p53 is activated progressively and the promotor selectivity of p53 is governed by its concentration. For repairable DNA damage, p53 accumulates to an intermediate level and induces p21 to arrest the cell cycle. For severe damage, p53 is driven to high levels, inducing Bax, PTEN and DDB2. Meanwhile, p21 is mediated by DDB2 to degrade markedly. Consequently, the inhibition of E2F1 is released, and apoptosis is then triggered. Therefore, p21 is regulated by p53 and DDB2 through an incoherent feed-forward loop, and its downregulation is essential for apoptosis induction.

## Materials and Methods

**Figure 1:**
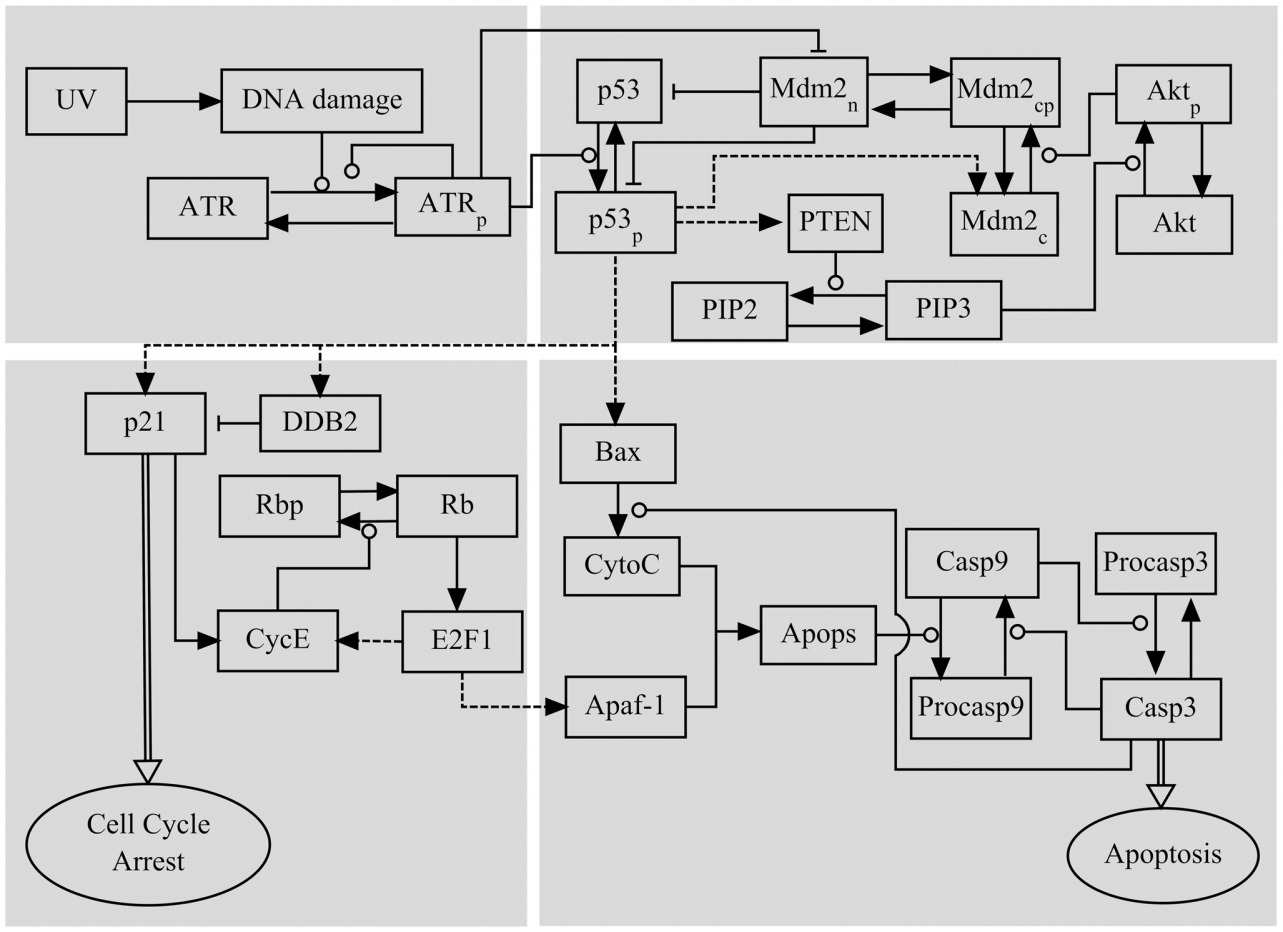
Schematic diagram of the model for the p53 network responding to UV-induced DNA damage. The model is composed of four modules. Two p53-centered feedback loops are considered: the p53-Mdm2 negative feedback loop and the p53-PTEN-Akt-Mdm2 positive feedback loop. Cell cycle arrest is induced through p21-dependent inhibition of E2F1, while apoptosis is triggered via the caspase cascade activated by Bax and Apaf-1. Dashed lines denote gene expression induced by p53 and E2F1. Solid arrowed lines represent transitions between proteins, promotion or inhibition of which is denoted by circle-headed and bar-headed lines, respectively.

The integrated model is composed of four modules, characterizing DNA repair and ATR activation, regulation of p53 activity, control of the cell cycle, and induction of apoptosis (Figure 1). Compared with our previous models [7]–[9], the current model characterizes ATR activity, the dual roles of p21 and its downregulation by DDB2 while ignoring the effect of p53 phosphorylation on its promoter selectivity for simplicity. Here, the selectivity depends on the concentration of p53. The concentration of each species is represented by a dimensionless state variable in rate equations ([…] denotes the concentration of species throughout the paper). These ordinary differential equations together with standard parameter values are presented in *Supporting Information*.

### DNA Repair and ATR Activation

UV radiation is generally divided into three wavebands: UVA (315–380 nm), UVB (280–315 nm), and UVC (190–280 nm) [24]. Most laboratories use short-wavelength UVC (254 nm) as an irradiation source in the study of UV-induced DNA damage and repair. Initially, two major lesions are induced by UVC radiation, i.e., CPDs and 6–4 PPs [18]. In mammalian cells, such damage is mainly fixed by the NER that is composed of four steps [25]. At the beginning, the damage is recognized by several NER factors like the XPC-hHR23B complex. Then, a larger region around the damage is opened up by transcription factor IIH. Subsequently, the damaged strand is cleaved by the XPG and ERCC1-XPF nucleases, inducing SSBs. Finally, a repair patch of about 30 nucleotides is produced in a DNA repair synthesis reaction and fills the excision gap. Thus, SSBs are produced rapidly due to DNA excision in the NER and are fixed slowly in the synthesis and ligation phase of NER [26].

For simplicity, the generation of SSBs was not characterized in our model. We paid attention to the process after the production of SSBs by DNA excision. The level of DNA damage is denoted by *L_D_*. For simplicity, SSBs are assumed to be fixed at a constant rate (Eq. 1). Single-strand DNA (ss-DNA) is coated with replication protein A (RPA) [27]. ATR is then recruited to the damage site by RPA-ssDNA in a complex with ATR-interacting protein (ATRIP) [28]. The recruitment of ATR-ATRIP enhances its congregation and promotes ATR phosphorylation at Thr1989. The phosphorylation further promotes engagement of the ATR-ATRIP complex and stimulates ATR by facilitating its substrate recognition [28]. Thus, ATR promotes its own activation by autophosphorylation on RPA-ssDNA.

We simplified the complicated process of ATR activation in the model. Two forms of ATR are considered: ATR (inactive form) and ATR_p_ (active form). The dynamics of ATR are described by Eqs. 3–4. The total level of ATR is assumed to be constant since ATR is mainly regulated posttranslationally [27]. The phosphorylation and dephosphorylation of ATR are characterized by the Michaelis-Menten kinetics [29]. The rate of ATR phosphorylation is assumed to be proportional to the level of ATR_p_. We set 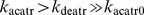 to ensure that most of ATR is inactive in unstressed cells and ATR is quickly activated upon UV radiation [27].

### Regulation of p53 Activity

Activated ATR induces the phosphorylation of p53 at Ser15 and Mdm2 at Ser407 [30], [31], leading to disruption of the p53-Mdm2 interaction and inhibition of the E3-ligase activity of Mdm2. As a result, p53 is stabilized and activated. Two forms of nuclear p53 are considered here: p53 (nonphosphorylated, inactive form) and p53_p_ (phosphorylated, active form). Mdm2 is divided into three forms: Mdm2_c_ (dephosphorylated cytoplasmic form), Mdm2_cp_ (phosphorylated cytoplasmic form), and Mdm2_n_ (nuclear form). We assume that only Mdm2_cp_ can enter the nucleus [32].

The p53-Mdm2 negative feedback loop and the p53-PTEN-Akt-Mdm2 positive feedback loop modulate p53 activity [33], [34]. p53 induces Mdm2, whereas Mdm2 inhibits p53 activity by promoting its degradation and repressing its transcriptional activity [33]. On the other hand, p53-induced PTEN inhibits Akt activity via PIP3 (phosphatidylinositol-3,4,5-trisphosphate) [35], [36], preventing the phosphorylation and nuclear entry of Mdm2_c_ [32]. The dynamics of this module are characterized by Eqs. 5–16. The expression of target genes by p53 is all characterized by a Hill function, and a Hill coefficient is set to 4 since p53 acts as a transcription factor in a tetrameric form [37]. The total level of Akt is assumed to be constant since no significant changes were observed in the DNA damage response [38]. Similarly, the total level of PIP3 and PIP2 (phosphatidylinositol-4, 5-bisphosphate) is also assumed to be constant.

It is worth noting that PTEN activity can be directly inhibited by PtdIns(3,4,5)P3-dependent Rac exchanger factor 2 (PREX2) [39]. PREX2 is a component of the PI3K/PTEN/Akt signaling pathway and antagonizes PTEN activity by protein-protein interaction [40]. PREX2 is frequently mutated and overexpressed in breast cancers with wild-type PTEN, and combined mutation of PREX2 and PIK3CA may lead to aberrant growth and transformation of cells [41]. Most recently, analysis of whole-genome sequence data identified PREX2 as a candidate melanoma gene, whose mutations appeared to undergo positive selection in human melanoma genesis [42]. Although PREX2 is implicated in UV-induced melanoma pathogenesis, the mechanism for PREX2 regulation following UV radiation is unknown. Therefore, we did not involve PREX2 in the present model, and its effect on cell-fate decision could be investigated when enough experimental data are available.

### Control of the Cell Cycle by the Rb/E2F1 Pathway

E2F1 plays an essential role in cell cycle progression [43]. In quiescent cells, E2F1 activity is inhibited by Rb. In the presence of growth factors like serum, upregulated cyclin D forms a complex with CDK4/6, leading to hyperphosphorylation of Rb and release of E2F1 [44]. Subsequently, E2F1 induces cyclin E (CycE), which binds CDK2 and further activates E2F1, leading to S-phase entry [45]. Due to the positive feedback between CycE induction and E2F1 activation, the G1/S transition can be characterized by a bistable switch [46]. Following DNA damage, however, p21 is induced by p53 and prevents E2F1 activation by inhibiting CDK2-cyclin E to arrest the cell cycle in G1 phase [47].

We characterized the control of cell cycle by the Rb/E2F1 pathway in a similar manner to that in [9] (Eqs. 17–26). Two forms of p21 are considered: p21 (free p21) and p21CE (p21 in complex with CDK2-cyclin E). The Rb protein may be in one of three states: Rb (free, non-phosphorylated Rb), Rb_p_ (free, hyperphosphorylated Rb), and RE (Rb in complex with E2F1). E2F1 exists in two forms: E2F1 (active free E2F1) and RE (inactive Rb-bound E2F1). The total levels of p21, cyclin E, Rb and E2F1 are denoted by [p21_tot_], [CycE_tot_], Rb_tot_ and E2F1_tot_, respectively. [p21_tot_] and [CycE_tot_] are determined by the levels of p53_p_ and free E2F1 at the transcriptional level, respectively. Rb_tot_ and E2F1_tot_ are assumed to be constant.

### Apoptosis Induction

p53-induced Bax contributes significantly to apoptosis induction [48]. Bax forms an oligomer at the outer membrane of mitochondria and promotes mitochondrial outer membrane permeabilization (MOMP) [49], resulting in the release of cytochrome c (CytoC). E2F1 also promotes apoptosis by inducing Apaf-1 [50]. CytoC and Apaf-1 assemble into an oligomeric apoptosome (Apops), which provides a platform for activation of caspase-9 (Casp9) [51]. Finally, caspase-3 (Casp3) is activated by Casp9 and apoptosis ensues [51]. Casp3 also promotes the activation of Casp9 [52]. In contrast to Bax and Apaf-1, p21 plays an inhibitory role in apoptosis induction [12]. The dynamics of this module are characterized by Eqs. (27–32).

We assume that p21 inhibits apoptosis via repressing expression of Apaf-1 indirectly, consistent with the recent observation that p21 blocks apoptosis downstream of mitochondria by inhibition of CDK-dependent Casp9 activation [14]. In contrast, p53-induced DDB2 contributes to apoptosis by promoting the degradation of p21 [23]. We assume that the degradation rate of p21 is positively correlated with the level of DDB2. To distinguish severe DNA damage from mild damage, we assume that the thresholds of transactivation for p53-induced proapoptotic genes (like PTEN, DDB2 and Bax) are higher than those for the proarrest genes (like p21), consistent with experimental observations [53].

### Methods

The rate equations were numerically solved using Oscill8 (http://oscill8.sourceforge.net). Time is in unit of minutes, while the units of parameters are determined such that the concentration of proteins is dimensionless. For simplicity, we did not provide the units of the parameters. The time step of integration was 0.01 min. All the initial values of variables were their lower steady-state values under unstressed conditions. Steady-state values of protein concentrations were obtained by setting the right-hand sides of ordinary differential equations to zero.

## Results

### Dynamics of Key Components of the p53 Network

**Figure 2:**
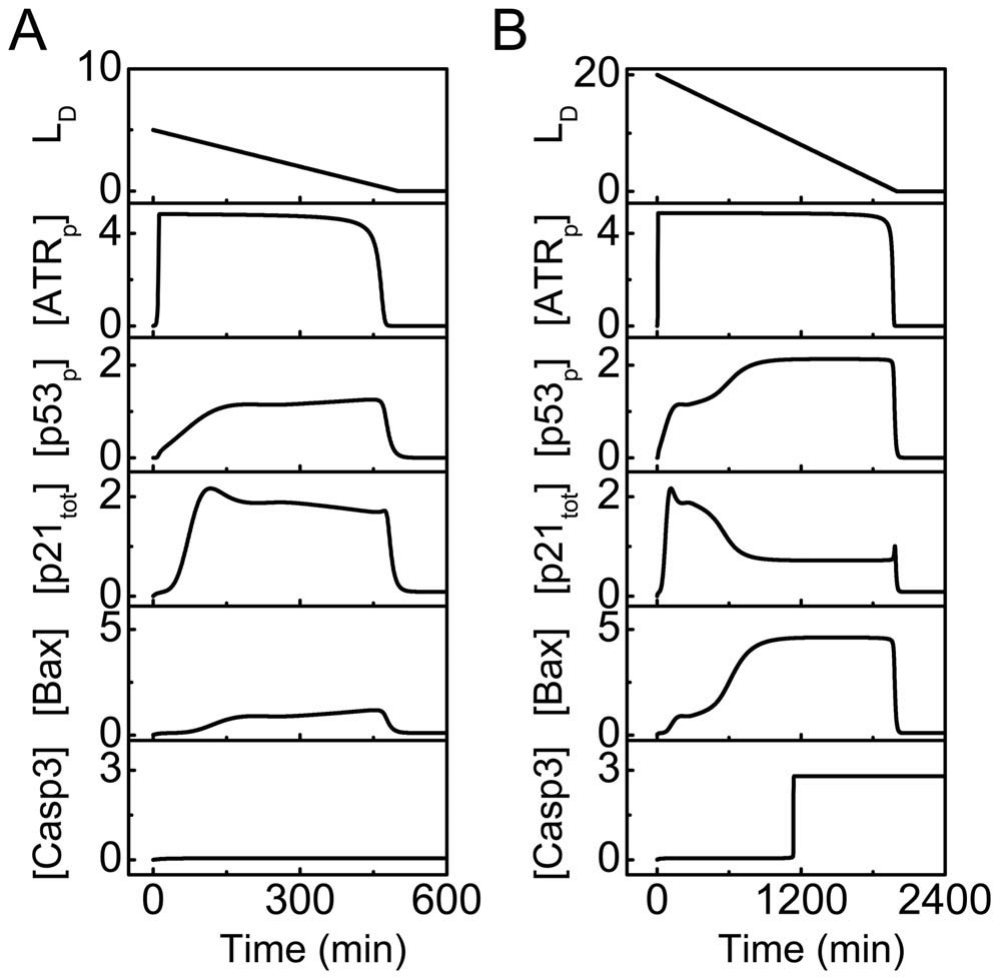
Dynamics of key components of the p53 network. Temporal evolution of the levels of DNA damage, ATR_p_, p53_p_, p21_tot_, Bax, and Casp3 (from top to bottom) at 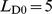 (A) or 20 (B).

We first show the dynamics of key components of the p53 network under typical stress conditions. The level of initial DNA damage is denoted by 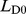. At 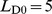, mild DNA damage is repairable. During the repair process, [ATR_p_] remains at a high level, while [p53_p_] rises to an intermediate level (Figure 2A). Consequently, p21 is induced to arrest the cell cycle, allowing for DNA repair, whereas [Bax] and [Casp3] remain at basal levels. Finally, the cell recovers to normal proliferation after the damage is fixed.

At 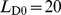, ATR remains active for a long time (Figure 2B). Differently, the dynamics of the other proteins exhibit a two-phase behavior. [p53_p_] first reaches an intermediate level and then rises to a higher level in the first phase; it remains there throughout the second phase. In the first phase, p21 is first expressed at a high level; later, [p21] drops gradually, whereas Bax accumulates markedly. In the second phase, [p21] and [Bax] are kept at low and high levels, respectively. Following full activation of Bax, Casp3 is activated in a switchlike manner, and apoptosis ensues. Note that we did not model the apoptotic events following Casp3 activation; otherwise, apoptosis should be committed before the DNA damage could be fixed. Taken together, moderate levels of p53 induce cell cycle arrest, while high levels of p53 trigger apoptosis. This is consistent with the observation that p53 shows a higher binding affinity for proarrest genes than for proapoptotic genes and sufficient p53 is required for apoptosis induction [53].

### Dynamics of ATR Activation

**Figure 3:**
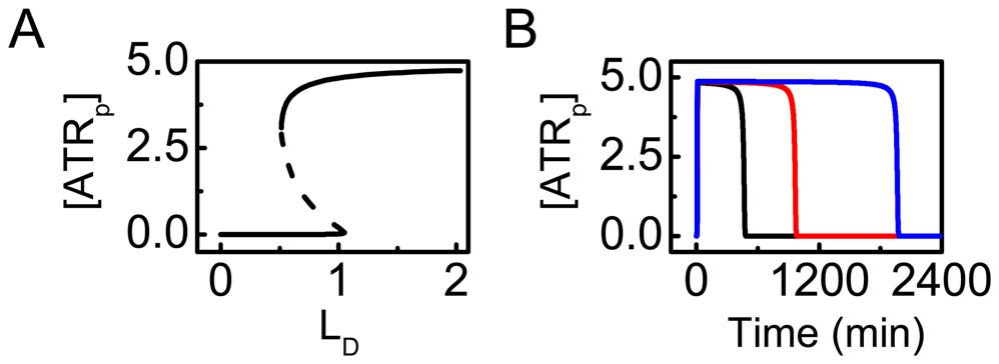
Switching behavior of ATR as a sensor of DNA damage. (A) Bifurcation diagram of [ATR_p_] vs. 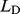 (without DNA repair). (B) Time courses of [ATR_p_] at 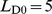 (black), 10 (red), or 20 (blue).

Here, we explore the mechanism for ATR activation upon DNA damage. As shown in Figure 2, [ATR_p_] undergoes a switchlike behavior. In the bifurcation diagram of steady-state level of ATR_p_ versus 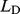, there exists a saddle-node bifurcation (Figure 3A). When 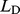 exceeds the upper threshold (1.1), [ATR_p_] switches to the “ON” state; only when 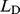 drops below the lower threshold (0.55), [ATR_p_] flips to the “OFF” state. Therefore, ATR activation behaves like a switch. In such a manner, ATR is both sensitive and reliable as a sensor of UV-induced DNA damage.

Figure 3B displays the time courses of [ATR_p_] at different damage levels. ATR is activated quickly upon DNA damage. The duration of ATR activation is proportional to the extent of the damage. Collectively, as a potent sensor of DNA damage, ATR is able to precisely transmit the damage signal downstream since the duration of ATR activation signifies the severity of the damage.

### Dynamics of p53 and its Regulation

**Figure 4:**
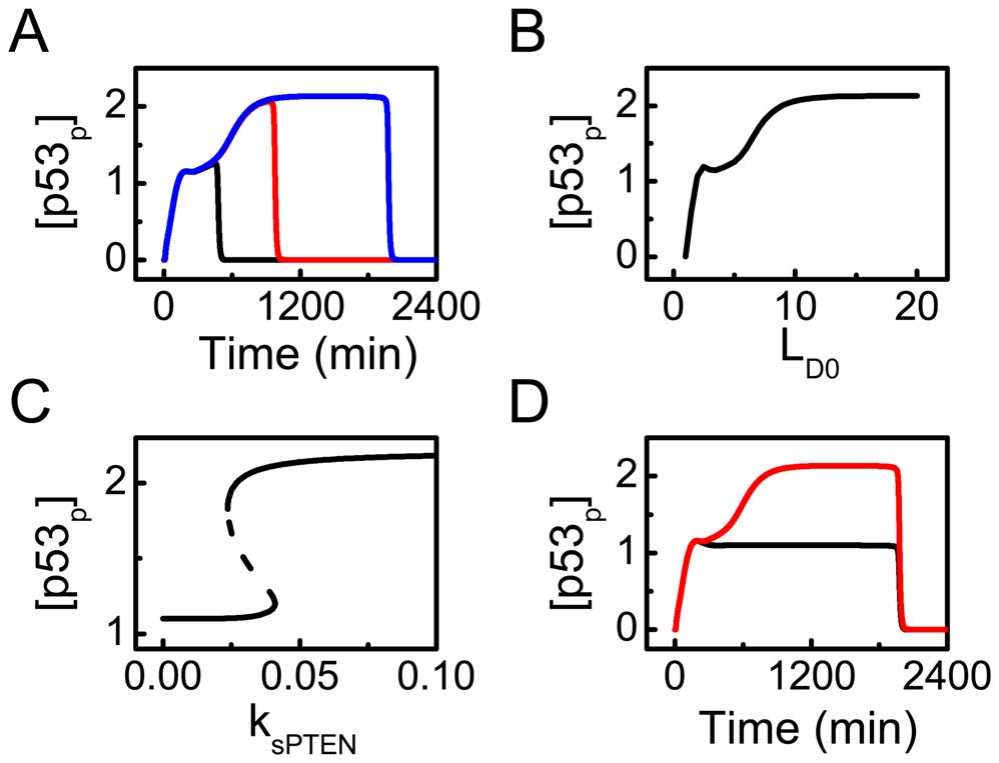
Role of PTEN in the two-phase dynamics of [p53_p_]. (A) Time courses of [p53_p_] at 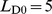 (black), 10 (red), or 20 (blue). (B) Maximum of [p53_p_] throughout the cellular response vs. 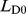. (C) Bifurcation diagram of [p53_p_] vs. the p53-inducible production rate of PTEN, 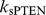, at fixed 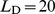. (D) Time courses of [p53_p_] with 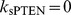 (black) or 0.05 (red).

It is intriguing to probe how p53-centered feedback loops regulate p53 dynamics. There are two feedback loops: the p53-Mdm2 loop and the p53-PTEN-Akt-Mdm2 loop. ATR_p_ activates p53 by phosphorylating p53 and Mdm2, disrupting the p53-Mdm2 loop. At 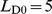, [p53_p_] can be kept at a moderate level (Figure 4A). At 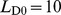, p53_p_ further accumulates, but its level drops sharply before reaching a plateau due to DNA repair. At 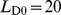, [p53_p_] can remain at a higher level for a longer period. Thus, the extent of initial DNA damage affects the level and duration of p53 activation.

The maximum level of p53_p_ in response to various levels of damage is shown in Figure 4B. The maximum first rises quickly with increasing 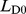 (since p53 accumulation is interrupted due to DNA repair). It then settles at moderate values for 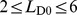. Subsequently, it further rises and finally remains at a plateau for 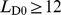. That is, p53 is activated progressively and becomes fully activated following severe damage. In general, disrupting the p53-Mdm2 loop is crucial for the primary activation of p53, and enhancing the p53-PTEN-Akt-Mdm2 loop promotes the full activation of p53 [34]. Specifically, PTEN contributes remarkably to p53 activation. PTEN inactivates Akt, leading to the sequestration of Mdm2 in the cytoplasm and upregulation of nuclear p53.

The above point is also reflected in the bifurcation diagram of steady-state level of p53_p_ versus the p53-inducible production rate of PTEN, 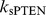 (Figure 4C). Of note, the diagram is obtained by keeping 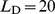 without DNA repair. [p53_p_] settles at a moderate level when 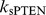 is lower than the upper threshold (0.041). After 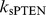 exceeds this threshold, [p53_p_] switches to higher levels. Thus, sufficient expression of PTEN is required to drive [p53_p_] to higher levels in severely damaged cells.

With 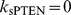, [p53_p_] indeed stays at a moderate level throughout the response even at 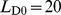 (Figure 4D). Thus, apoptosis cannot be evoked even in severely damage cells. PTEN deficiency may result in senescence in irreparably damaged cells [54]. Collectively, the p53-PTEN-Akt-Mdm2 loop amplifies p53 accumulation during the late phase of the response, and PTEN has a crucial role in fully activating p53 [34].

### p21-induced Cell Cycle Arrest and Apoptosis Inhibition

**Figure 5:**
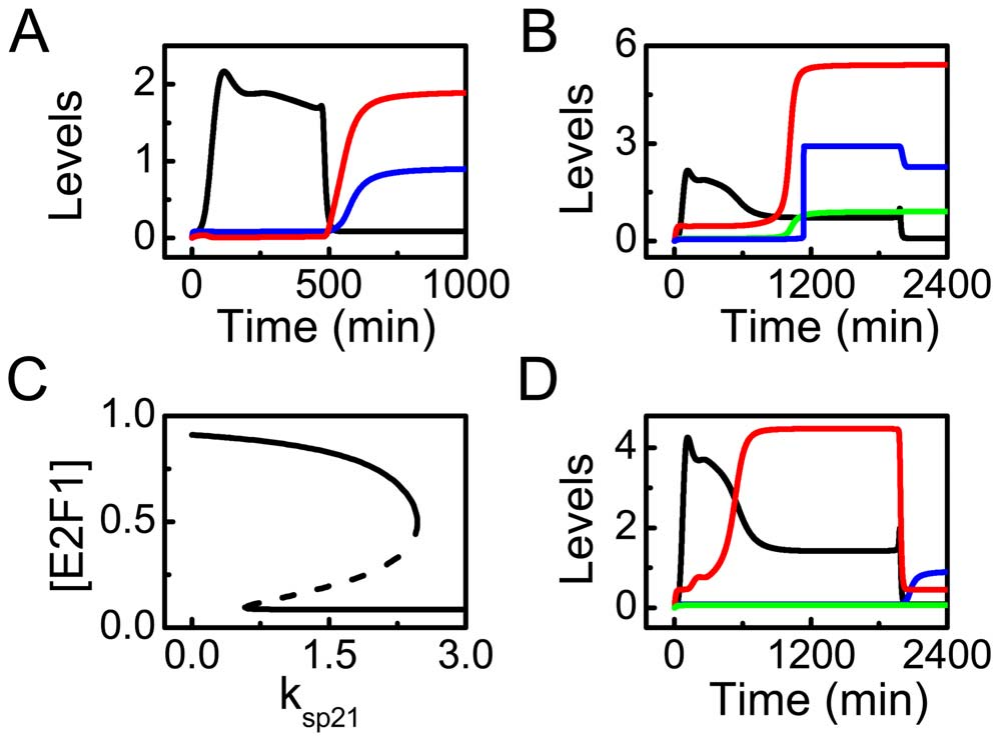
Regulation of cell cycle progression and apoptosis by p21. (A) Time courses of [p21_tot_] (black), [Rb_p_] (red), and [E2F1] (blue) at 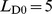. (B) Time courses of [p21_tot_] (black), [E2F1] (green), [Apaf-1] (red), and [Casp9] (blue) at 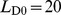. (C) Bifurcation diagram of [E2F1] vs. the p53-inducible production rate of p21, 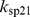. (D) Time courses of [p21_tot_] (black), [E2F1] (blue), [CytoC] (red), and [Casp9] (green) with 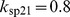 and 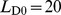.

p21 is known as a CDK inhibitor, inhibiting the activity of CDK2-cyclin E [11]. Thus, p21 activity is antagonistic to E2F1 activity. Upon DNA damage of 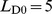, [p21_tot_] rises quickly and remains at a relatively high level before dropping to a basal level (Figure 5A). [Rb_p_] remains at a low level during the DNA repair process since the phosphorylation of Rb via CDKs is inhibited by p21. Accordingly, [E2F1] is low due to repression by Rb. Therefore, high levels of p21 induce cell cycle arrest. After the damage is fixed, E2F1 is reactivated, and the cell resumes proliferation.

Besides arresting the cell cycle, p21 also plays an inhibitory role in apoptosis induction. The dynamics of [p21_tot_], [E2F1] and [Apaf-1] and [Casp9] at 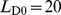 are displayed in Figure 5B. The temporal evolution can be divided into two phases. In the first phase, [p21_tot_] first rises and then falls gradually. Meanwhile, the other proteins remain at low levels. In the second phase, [p21] is kept at low levels. Consequently, the inhibition of E2F1 is released, and activated E2F1 induces Apaf-1. Later, Casp9 is activated to trigger apoptosis. Therefore, p21 prevents apoptosis by inhibiting E2F1, and downregulation of p21 is required for apoptosis induction.

To further explore the effect of p21 expression on E2F1 activation, we plot the bifurcation diagram of steady-state level of E2F1 (at fixed 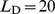) versus 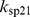 (Figure 5C). [E2F1] exhibits bistability for 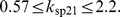 [E2F1] settles in the upper state when 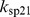 is below the lower threshold (0.57). That is, E2F1 can be activated to promote apoptosis at 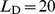 when p21 has a low expression rate. When 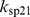 exceeds the upper threshold (2.2), [E2F1] always stays at low levels. Therefore, overexpression of p21 may prevent irreparable cells from apoptosis by inhibiting E2F1.

At 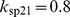, p21 is overexpressed; its maximum level is nearly twice that at 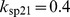 (the standard value) (Figure 5D). Although [p21_tot_] still decreases after its induction, it is high enough in the second phase so that E2F1 is totally inhibited. Consequently, Casp9 remains inactive although a large amount of CytoC is released from mitochondria. These results agree well with the observation that p21 can inhibit apoptosis downstream of mitochondria by preventing Casp9 activation [14], suggesting that p21 is also an important node controlling apoptosis. Therefore, p21 has a dual role, arresting the cell cycle and repressing apoptosis. This may represent a robust mechanism, contributing to cell survival and ensuring that apoptosis is committed only in severely damaged cells.

### DDB2 Promotes Apoptosis Induction

**Figure 6:**
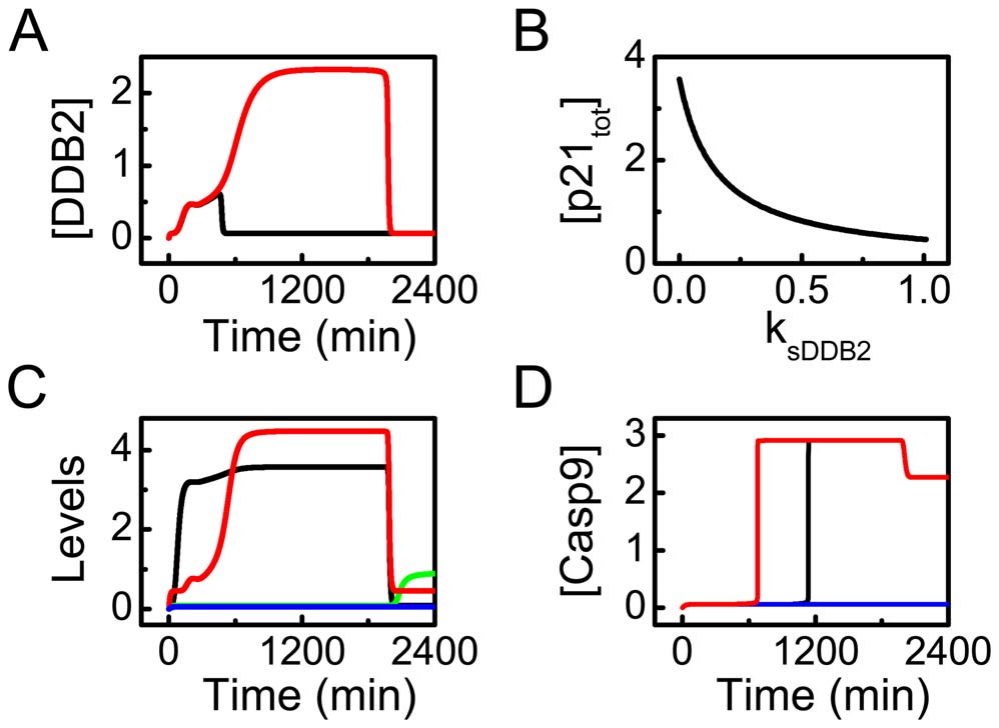
DDB2 promotes apoptosis via mediating the degradation of p21. (A) Time courses of [DDB2] at 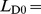 5 (black) or 20 (red). (B) Bifurcation diagram of [p21_tot_] vs. the p53-inducible production rate of DDB2, 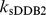. (C) Time courses of [p21_tot_] (black), [E2F1] (green), [Bax] (red), and [Casp9] (blue) with 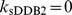 at 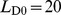. (D) Time courses of [Casp9] with the normal parameter setting (i.e., 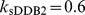 and 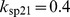) (black), 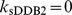 and 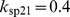 (blue), or 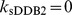 and 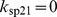 (red).

We have revealed that p21 is an inhibitor of apoptosis and should be downregulated to trigger apoptosis. This can be accomplished by DDB2-mediated degradation of p21 [23]. The dynamics of [DDB2] are displayed in Figure 6A. Since DDB2 is directly induced by p53, its temporal evolution follows that of [p53_p_] (cf. Figure 4A). At 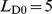, only low levels of DDB2 accumulate. At 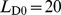, [DDB2] settles at a high level in the second phase of the response. It is evident that the accumulation of DDB2 leads to reduction of p21 and remarkable expression of DDB2 appears only in response to severe damage.

The bifurcation diagram of [p21_tot_] versus the p53-inducible production rate of DDB2, 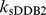, is presented to show the effect of DDB2 expression on p21 level (Figure 6B). The diagram is obtained at fixed 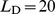. [p21_tot_] first falls monotonically with increasing 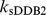 and tends to saturation when 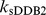 is rather large. Therefore, DDB2 expression significantly influences the downregulation of p21, consistent with the experimental observation in Ref. [23].

Figure 6C displays the dynamics of [p21_tot_], [E2F1], [CytoC] and [Casp9] with 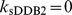 at 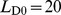. [p21_tot_] stays at a high level throughout the response, and E2F1 is persistently inhibited. As a result, Casp9 is inactivated even in the presence of CytoC release. Therefore, without degradation by DDB2, p21 prevents cells from apoptosis via inhibiting Apaf-1 expression by E2F1, which is regulated by CDKs activity [14]. These results also indicate that the downregulation of p21 is essential for apoptosis induction.

To further identify the role of DDB2 in apoptosis induction, we compare the dynamics of [Casp9] at 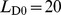 in three different cases. With the standard parameter values, Casp9 is activated in a switchlike manner (Figure 6D). But Casp9 is inactivated in DDB2 knockout cells (with 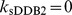) since they become resistant to severe damage (see also Figure 6C). When both DDB2 and p21 are knocked out, Casp9 is activated, i.e., apoptosis can be induced. Moreover, Casp9 activation is accelerated in this double-knockout case. These results show good agreement with the observation that deletion of p21 restores apoptosis in DDB2-knockout cells [23]. Thus, DDB2 promotes apoptosis via degrading p21, and p21 depletion makes cells more sensitive to DNA damage. Taken together, DDB2 plays a critical role in the cell-fate decision between survival and death by promoting the degradation of p21.

## Discussion

We developed an integrated model of the p53 network to explore the cell-fate decision after UV radiation, focusing on the dual roles of p21. We found that p53 can be activated progressively so that its target genes are selectively induced. For mild DNA damage, p53 rises to moderate levels and becomes primarily activated. p21 is induced to arrest the cell cycle, allowing for DNA repair. For severe damage, PTEN is induced to drive p53 to high levels, leading to its full activation. High levels of p53 enable remarkable expression of the proapoptotic genes such as Bax and DDB2. Bax induces the release of cytochrome c. DDB2 promotes the degradation of p21, releasing the inhibition of E2F1. Subsequently, E2F1-induced Apaf-1 and cytochrome c form the apoptosome that acts as a platform for caspase-9 activation. Finally, caspase-3 is activated, and apoptosis ensues. Remarkably, DDB2-mediated p21 degradation is crucial for the activation of caspase-9 downstream of mitochondrial cytochrome c release. Our results suggest that the coordination between p21 and DDB2 has an important role in determining cellular outcome.

Besides as a potent inhibitor of cell cycle progression, p21 inhibits apoptosis induction [12]. Given its dual roles in cell-fate decision, the regulation of p21 activity becomes important for an appropriate cellular response to DNA damage. Under most conditions, p21 is transactivated by p53 to arrest the cell cycle [10]. To induce apoptosis, p21 should be downregulated. There exist several mechanisms for p21 downregulation, and which mechanism is at work may depend on cellular context and stress types. Our model focused on the degradation of p21 mediated by DDB2 following UV-induced DNA damage [23]. p21 can also be downregulated via transcriptional repression. The p53-induced Hzf acts as a cofactor for transactivation of p21 by p53 [55]. Following severe damage caused by ionizing radiation, Hzf is degraded by an unknown E3-ligase, leading to a reduction in p21 transcription [55]. A theoretical model was built to clarify the underlying mechanism in that case [9]. Although p21 downregulation facilitates apoptosis induction, the complete deletion of p21 may lead to aberrant cell proliferation even after irreparable damage [56]. In the present work, p21 induces cell cycle arrest upon mild damage, while p21 is downregulated by DDB2 to facilitate apoptosis induction in the late phase of the response to severe damage. Such elaborate dynamics of p21 reconcile its dual role in cell cycle control and apoptosis regulation.

DDB2 was first found involved in the NER, which is engaged in the repair of UV-induced DNA damage [20]. It was found later that DDB2 also acts as an adapter protein linking ubiquitination substrates to the E3 ligase complex, such as Cul4-DDB1 [22]. For the sake of simplicity, the role of DDB2 in DNA repair was neglected in the model. DDB2 also has a dual role in the DNA damage response, and its role in the NER seems contradictory to its role in apoptosis induction. When more experimental data are available, a detailed model could be proposed to reconcile the dual roles of DDB2 in the regulation of NER and apoptosis induction.

## Supporting Information

Materials S1 **Ordinary differential equations and standard parameter values for the model.**
(PDF)
