# Supplementary material for: Coordination between p21 and DDB2 in the Cellular Response to UV Radiation
Source: PLoS One. 2013 Nov 18;8(11):e80111. doi: 10.1371/journal.pone.0080111 (PMC3832521; doi:10.1371/journal.pone.0080111)
Supplement: Materials S1 — Ordinary differential equations and standard parameter values for the model. (PDF) [file pone.0080111.s001.pdf]

SUPPORTING MATERIAL

Coordination between p21 and DDB2 in the Cellular Response to UV Radiation

Hao Li, Xiao-Peng Zhang, Feng Liu

Here, we present the ordinary differential equations for the model and standard parameter values.

I: Ordinary Differential Equations for the Model

$$\frac{dL_{\mathrm{D}}}{dt} = -k_{\mathrm{repair}}\mathrm{H}(L_{\mathrm{D}}) \tag{1}$$

$$H(x) = \begin{cases} 1 & x \geq 0 \\ 0 & x < 0 \end{cases} \tag{2}$$

$$\frac{d[\mathrm{ATR_p}]}{dt} = \left(k_{\mathrm{acatr0}} + k_{\mathrm{acatr}}\frac{L_{\mathrm{D}}}{L_{\mathrm{D}} + j_{\mathrm{d}}}[\mathrm{ATR_p}]\right)\frac{[\mathrm{ATR}]}{[\mathrm{ATR}] + j_{\mathrm{acatr}}} - k_{\mathrm{deatr}}\frac{[\mathrm{ATR_p}]}{[\mathrm{ATR_p}] + j_{\mathrm{deatr}}} \tag{3}$$

$$[\mathrm{ATR}] = \mathrm{ATR_{tot}} - [\mathrm{ATR_p}] \tag{4}$$

$$k_{\mathrm{acp53}} = k_{\mathrm{acp530}}\frac{[\mathrm{ATR_p}]}{[\mathrm{ATR_p}] + j_{\mathrm{atr}}} \tag{5}$$

$$\frac{d[\mathrm{p53}]}{dt} = k_{\mathrm{sp53}} - k_{\mathrm{dp530}}[\mathrm{p53}] - k_{\mathrm{dp53}}[\mathrm{Mdm2_n}]\frac{[\mathrm{p53}]}{[\mathrm{p53}] + j_{\mathrm{dp53}}} - k_{\mathrm{acp53}}[\mathrm{p53}] + k_{\mathrm{dep53}}[\mathrm{p53_p}] \tag{6}$$

$$\frac{d[\mathrm{p53_p}]}{dt} = k_{\mathrm{acp53}}[\mathrm{p53}] - k_{\mathrm{dep53}}[\mathrm{p53_p}] - k_{\mathrm{dp53p}}[\mathrm{Mdm2_n}]\frac{[\mathrm{p53_p}]}{[\mathrm{p53_p}] + j_{\mathrm{dp53p}}} \tag{7}$$

$$\frac{d[\mathrm{Mdm2_c}]}{dt} = k_{\mathrm{smdm20}} + k_{\mathrm{smdm2}}\frac{[\mathrm{p53_p}]^4}{[\mathrm{p53_p}]^4 + j_{\mathrm{smdm2}}^4} - k_{\mathrm{dmdm2}}[\mathrm{Mdm2_c}] + k_{\mathrm{dpmdm2}}\frac{[\mathrm{Mdm2_{cp}}]}{[\mathrm{Mdm2_{cp}}] + j_{\mathrm{dpmdm2}}}$$
$$-k_{\mathrm{pmdm2}}[\mathrm{Akt_p}]\frac{[\mathrm{Mdm2_c}]}{[\mathrm{Mdm2_c}] + j_{\mathrm{pmdm2}}} \tag{8}$$

$$\frac{d[\mathrm{Mdm2_{cp}}]}{dt} = k_{\mathrm{pmdm2}}[\mathrm{Akt_p}]\frac{[\mathrm{Mdm2_c}]}{[\mathrm{Mdm2_c}] + j_{\mathrm{pmdm2}}} - k_{\mathrm{dpmdm2}}\frac{[\mathrm{Mdm2_{cp}}]}{[\mathrm{Mdm2_{cp}}] + j_{\mathrm{dpmdm2}}} - k_{\mathrm{inmdm2c}}[\mathrm{Mdm2_{cp}}]$$
$$+k_{\mathrm{outmdm2n}}[\mathrm{Mdm2_n}] - k_{\mathrm{dmdm2}}[\mathrm{Mdm2_{cp}}] \tag{9}$$

$$\frac{d[\mathrm{Mdm2_n}]}{dt} = k_{\mathrm{inmdm2c}}[\mathrm{Mdm2_{cp}}] - k_{\mathrm{outmdm2n}}[\mathrm{Mdm2_n}] - (k_{\mathrm{dmdm2}} + k_{\mathrm{dmdm2n}})[\mathrm{Mdm2_n}] \tag{10}$$

$$k_{\mathrm{dmdm2n}} = k_{\mathrm{dmdm2n0}}\frac{[\mathrm{ATR_p}]}{[\mathrm{ATR_p}] + j_{\mathrm{atr}}} \tag{11}$$

$$
\begin{aligned}
\frac{d[\mathrm{Akt_p}]}{dt} &= k_{\mathrm{pakt}}[\mathrm{PIP3}]\frac{[\mathrm{Akt}]}{[\mathrm{Akt}]+j_{\mathrm{pakt}}} - k_{\mathrm{dpakt}}\frac{[\mathrm{Akt_p}]}{[\mathrm{Akt_p}]+j_{\mathrm{dpakt}}} &(12)\\
[\mathrm{Akt}] &= \mathrm{Akt_{tot}} - [\mathrm{Akt_p}] &(13)\\
\frac{d[\mathrm{PIP3}]}{dt} &= k_{\mathrm{p2}}\frac{[\mathrm{PIP2}]}{[\mathrm{PIP2}]+j_{\mathrm{p2}}} - k_{\mathrm{p3}}[\mathrm{PTEN}]\frac{[\mathrm{PIP3}]}{[\mathrm{PIP3}]+j_{\mathrm{p3}}} &(14)\\
[\mathrm{PIP2}] &= \mathrm{PIP_{tot}} - [\mathrm{PIP3}] &(15)\\
\frac{d[\mathrm{PTEN}]}{dt} &= k_{\mathrm{sPTEN0}} + k_{\mathrm{sPTEN}}\frac{[\mathrm{p53_p}]^4}{[\mathrm{p53_p}]^4 + j_{\mathrm{sPTEN}}^4} - k_{\mathrm{dPTEN}}[\mathrm{PTEN}] &(16)\\
\frac{d[\mathrm{DDB2}]}{dt} &= k_{\mathrm{sDDB20}} + k_{\mathrm{sDDB2}}\frac{[\mathrm{p53_p}]^4}{[\mathrm{p53_p}]^4 + j_{\mathrm{sDDB2}}^4} - k_{\mathrm{dDDB2}}[\mathrm{DDB2}] &(17)\\
\frac{d[\mathrm{p21_{tot}}]}{dt} &= k_{\mathrm{sp210}} + k_{\mathrm{sp21}}\frac{[\mathrm{p53_p}]^4}{[\mathrm{p53_p}]^4 + j_{\mathrm{sp21}}^4} - k_{\mathrm{dp210}}[\mathrm{p21_{tot}}] - k_{\mathrm{dp21}}[\mathrm{DDB2}][\mathrm{p21_{tot}}] &(18)\\
\frac{\mathrm{d}[\mathrm{p21CE}]}{\mathrm{dt}} &= k_{\mathrm{asp21ce}}[\mathrm{p21}][\mathrm{CycE}] - k_{\mathrm{dsp21ce}}[\mathrm{p21CE}] &(19)\\
[\mathrm{p21}] &= [\mathrm{p21_{tot}}] - [\mathrm{p21CE}] &(20)\\
\frac{d[\mathrm{CycE_{tot}}]}{dt} &= k_{\mathrm{scyce0}} + k_{\mathrm{scyce}}\frac{[\mathrm{E2F1}]^2}{[\mathrm{E2F1}]^2 + j_{\mathrm{scyce}}^2} - k_{\mathrm{dcyce}}[\mathrm{CycE_{tot}}] &(21)\\
[\mathrm{CycE}] &= [\mathrm{CycE_{tot}}] - [\mathrm{p21CE}] &(22)\\
\frac{d[\mathrm{E2F1}]}{dt} &= -k_{\mathrm{asre}}[\mathrm{Rb}][\mathrm{E2F1}] + k_{\mathrm{dsre}}[\mathrm{RE}] &(23)\\
[\mathrm{RE}] &= \mathrm{E2F1_{tot}} - [\mathrm{E2F1}] &(24)\\
\frac{d[\mathrm{Rb_p}]}{dt} &= k_{\mathrm{prb}}[\mathrm{CycE}]\frac{[\mathrm{Rb}]}{[\mathrm{Rb}]+j_{\mathrm{prb}}} - k_{\mathrm{dprb}}\frac{[\mathrm{Rb_p}]}{[\mathrm{Rb_p}]+j_{\mathrm{dprb}}} &(25)\\
[\mathrm{Rb}] &= \mathrm{Rb_{tot}} - [\mathrm{Rb_p}] - [\mathrm{RE}] &(26)\\
\frac{d[\mathrm{Bax}]}{dt} &= k_{\mathrm{sbax0}} + k_{\mathrm{sbax}}\frac{[\mathrm{p53_p}]^4}{[\mathrm{p53_p}]^4 + j_{\mathrm{sbax}}^4} - k_{\mathrm{dbax}}[\mathrm{Bax}] &(27)\\
\frac{d[\mathrm{CytoC}]}{dt} &= \left(k_{\mathrm{accytoc0}} + k_{\mathrm{accytoc}}\frac{[\mathrm{Bax}]^4}{[\mathrm{Bax}]^4 + j_{\mathrm{bax}}^4}\right)(\mathrm{CytoC_{tot}} - [\mathrm{CytoC}]) - k_{\mathrm{decytoc}}[\mathrm{CytoC}] &(28)\\
\frac{d[\mathrm{Apaf1}]}{dt} &= k_{\mathrm{sapaf10}} + k_{\mathrm{sapaf1}}\frac{[\mathrm{E2F1}]^2}{[\mathrm{E2F1}]^2 + j_{\mathrm{sapaf1}}^2} - k_{\mathrm{dapaf1}}[\mathrm{Apaf1}] &(29)\\
\frac{d[\mathrm{Apops}]}{dt} &= k_{\mathrm{acapops}}\left(([\mathrm{CytoC}] - 7[\mathrm{Apops}])([\mathrm{Apaf1}] - 7[\mathrm{Apops}])\right)^7 - k_{\mathrm{deapops}}[\mathrm{Apops}] &(30)\\
\frac{d[\mathrm{Casp9}]}{dt} &= \left(k_{\mathrm{accasp90}} + k_{\mathrm{accasp9}}\frac{[\mathrm{Casp3}]^4}{[\mathrm{Casp3}]^4 + j_{\mathrm{casp3}}^4}\right)(\mathrm{Casp9_{tot}} - [\mathrm{Casp9}]) - k_{\mathrm{decasp9}}[\mathrm{Casp9}] &(31)\\
\frac{d[\mathrm{Casp3}]}{dt} &= \left(k_{\mathrm{accasp30}} + k_{\mathrm{accasp3}}\frac{[\mathrm{Casp9}]^4}{[\mathrm{Casp9}]^4 + j_{\mathrm{casp9}}^4}\right)(\mathrm{Casp3_{tot}} - [\mathrm{Casp3}]) - k_{\mathrm{decasp3}}[\mathrm{Casp3}] &(32)
\end{aligned}
$$

II: Standard Parameter Values

Parameter	Value	Parameter	Value	Parameter	Value	Parameter	Value	Parameter	Value
k_{repair}	0.01	k_{acatr0}	0.001	k_{acatr}	2.0	j_{acatr}	1.0	k_{deatr}	1.5
j_{deatr}	2.5	j_{d}	2.0	j_{atr}	1.0	ATR_{tot}	5.0	k_{sp53}	0.04
k_{dp530}	0.03	k_{dp53}	0.2	j_{dp53}	0.1	k_{dp53p}	0.01	j_{dp53p}	0.1
k_{acp530}	0.2	k_{dep53}	0.1	k_{dmdm2}	0.009	k_{smdm20}	0.002	k_{smdm2}	0.01
j_{smdm2}	1.0	k_{pmdm2}	4.0	j_{pmdm2}	0.3	k_{dpmdm2}	0.3	j_{dpmdm2}	0.1
k_{inmdm2c}	0.06	k_{outmdm2n}	0.09	k_{dmdm2n0}	0.05	k_{pakt}	0.25	k_{dpakt}	0.1
j_{pakt}	0.1	j_{dpakt}	0.2	Akt_{tot}	1.0	PIP_{tot}	1.0	k_{p2}	0.1
k_{p3}	0.45	j_{p2}	0.2	j_{p3}	0.4	k_{sPTEN0}	0.001	k_{sPTEN}	0.05
j_{sPTEN}	2.0	k_{dPTEN}	0.01	k_{sDDB20}	0.01	k_{sDDB2}	0.6	j_{sDDB2}	2.0
k_{dDDB2}	0.15	k_{sp210}	0.01	k_{sp21}	0.4	j_{sp21}	0.7	k_{dp210}	0.1
k_{dp21}	0.2	k_{scyce0}	0.0005	k_{scyce}	0.0275	j_{scyce}	0.2	k_{dcyce}	0.005
k_{asre}	0.5	k_{dsre}	0.05	k_{prb}	0.05	j_{prb}	0.1	k_{dprb}	0.025
j_{dprb}	0.1	k_{asp21ce}	0.5	k_{dsp21ce}	0.05	Rb_{tot}	2.0	E2F1_{tot}	1.0
k_{sbax0}	0.01	k_{sbax}	0.8	j_{sbax}	2.0	k_{dbax}	0.1	j_{bax}	3.0
k_{accytoc0}	0.01	k_{accytoc}	1.0	k_{decytoc}	0.1	k_{sapaf10}	0.001	k_{sapaf1}	0.6
j_{sapaf1}	0.6	k_{dapaf1}	0.1	k_{acapops}	5.0	k_{deapops}	0.5	k_{accasp90}	0.001
k_{accasp9}	3.0	j_{casp3}	0.5	k_{decasp9}	0.05	k_{accasp30}	0.001	k_{accasp3}	0.1
j_{casp9}	0.5	k_{decasp3}	0.07	$\text{CytoC}_{\text{tot}}$	5.0	$\text{Casp9}_{\text{ tot}}$	3.0	$\text{Casp3}_{\text{tot}}$	3.0
